# The Porcelain Inlay

**Published:** 1912-07-15

**Authors:** N. S. Jenkins


### THE PORCELAIN INLAY.

BY N. S. JENKINS, D.D.S.

No American dentist in Europe obtains a solid practice except through merit of high order. Everything else may be forgiven him except lack of skill, and therefore it has been that, from the beginning, porcelain work has been mastered and successfully practiced among the men who constitute this Society. But we also owe much to the European clientelle. Proud as we are of the great intellectual, political and material achievements of American civilization, we must yet admit that, in such particulars as matters of good taste, and in the general influence of the fine arts and the graces of society, we still defer to Europe. Among the accepted principles of European society is the rule that good taste must not be strikingly violated; but a conspicuous example of departure from this rule is found in the shocking dental disfiguration of great numbers of American patients. Such disfigurement, it is safe to say, occurs almost exclusively in America. I recently learned, in visiting the post-graduate school in Berlin, that it was difficult to find, even among the gratuitously treated patients, any who would allow themselves to be made hideous by visible gold fillings in the front teeth. Therefore, I repeat, we owe much to the aesthetic sense of our European clientelle that we have been able to keep our standard of practice, despite the temptation of the gold inlay, from such degradation. For a degradation it is that, in a land where dentistry has attained such practical and scientific development, and where, some fifteen years ago, there was an enthusiastic acceptance of the porcelain inlay, there has followed, to so great an extent, a relapse to an era of "barbaric gold and pearl." Those of us who have visited America during the past few years have been amazed to find, in all classes of American society, evidences of such disregard of aesthetic considerations as would be inconceivable in any European community.

It is no excuse that many American practitioners have never given themselves the trouble to learn the principles of porcelain work because their patients have not demanded it. The duty of every educated man is to lead, not to follow, and he who deliberately disfigures the poorest of his fellows rather than give himself the trouble of learning how to conceal his art, dishonors himself as much as his profession.

### GLASS BURNISHERS.

Some three years ago, Dr. Weber presented his system of burnishing the matrix with glass instruments to the American Dental Club of Paris. It is such a simple and effective device for obtaining a perfect matrix, that one wonders it was not thought of long ago. One takes a glass rod and holds one end over a Bunsen burner until the glass is softened, and then draws it out to the desired thinness and breaks it off. The broken end is then held over the flame again until the point melts and rolls up into a ball of the desired size. The shank can be given any desired curve by holding it over the flame until it softens.

Either in the mouth, or upon a model of the cavity, the foil can be burnished to absolute perfection. Take even a matrix which has been stamped to seemingly an exact fit, lay it upon the model and go over it with a glass burnisher, and you will find how completely the least wrinkle can be obliterated and how much more perfectly it can be made to fit. There is no longer a necessity for employing a press. More quickly and with greater accuracy, foil, especially gold foil, can in this manner be adapted to any cavity. Even before the advent of the glass burnisher, the more exact fit of the gold matrix led to its extended use, especially in Europe, where it has never lost its supremacy. With the glass burnisher its superiority is even more pronounced, it being possible to obliterate every fold or wrinkle and to give a smoothness and rigidity which can be obtained in no other way. The gold foil strips off in one piece, leaving the completed inlay a seemingly absolute fit. When such an inlay has been grooved and set in the cavity with an appropriate cement, it leaves no line which, even years afterward, can be detected.

In my visit to the post-graduate school in Berlin, Mamlok, the instructor in porcelain inlays, called in various patients who had been treated with porcelain restorations by the students during the past two years, within which time burnishing gold matrices with glass instruments had been taught. Among them all I could not discover a single dark line, or any evidence of edge defect. When we consider that these numerous operations had been made by practitioners from over all northern Europe, male and female dentists who had come for the purpose of improvement, and that they had all learned to make what seemed to be absolutely perfect porcelain restorations ever since this method had been taught, it is evident that we owe a debt of gratitude to Weber for this simple but most important and practical discovery.

### KORBITZ ELECTRIC FURNACE.

The second point in the vertical electric furnace of Korbitz. In my earliest experiments in fusing inlays, I was convinced of the superiority of the gold matrix, whose possibilities were first discovered by Sachs. It was evident that this matrix, and indeed it is also true of the platinum matrix, needed to be imbedded, and that the porcelain should be melted through heat applied from the bottom, that the contour might be the last to fuse and so the shape be more easily preserved. Therefore I made the gas furnace with the foot bellows, throwing the heat upon the bottom of the melting cup, with an opening for purposes of observation. This furnace has been useful in its day, for, although requiring considerable experiecne to work it properly, it was founded upon correct principles. It has remained for Korbitz, however, to give us now an electric furnace which is wired chiefly at the bottom. This vertical furnace is of chamotte, with a swinging chamotte cap, which can be used for drying the melting cup and afterwards be easily opened for purposes of observation during the process of fusing, or it may be left open during the entire process. As the heat comes chiefly from the bottom, the porcelain melts first in the lower part of the matrix and flows like wax into its position. At the last melting it should be carefully watched, and with a touch the process can be suspended exactly when the contour is as desired.

By these two methods the technique of making porcelain inlays is not only simplified, by the results thus obtained seem to insure that this beautiful art may now be practiced with greater ease and with scientific accuracy.—*Review*, July 1912.

